# Experimental sexual selection affects the evolution of physiological and life‐history traits

**DOI:** 10.1111/jeb.14003

**Published:** 2022-04-05

**Authors:** Martin D. Garlovsky, Luke Holman, Andrew L. Brooks, Zorana K. Novicic, Rhonda R. Snook

**Affiliations:** ^1^ Department of Animal and Plant Sciences University of Sheffield Sheffield UK; ^2^ School of Applied Sciences Edinburgh Napier University Edinburgh UK; ^3^ 8097 Animal Ecology, Department of Ecology and Genetics Evolutionary Biology Center Uppsala University Uppsala Sweden; ^4^ 7675 Department of Zoology Stockholm University Stockholm Sweden; ^5^ Present address: 9169 Applied Zoology Faculty Biology Technische Universität Dresden Dresden Germany

**Keywords:** experimental evolution, life‐history evolution, metabolism, physiology, polyandry, sexual selection, trade‐offs

## Abstract

Sexual selection and sexual conflict are expected to affect all aspects of the phenotype, not only traits that are directly involved in reproduction. Here, we show coordinated evolution of multiple physiological and life‐history traits in response to long‐term experimental manipulation of the mating system in populations of *Drosophila pseudoobscura*. Development time was extended under polyandry relative to monogamy in both sexes, potentially due to higher investment in traits linked to sexual selection and sexual conflict. Individuals (especially males) evolving under polyandry had higher metabolic rates and locomotor activity than those evolving under monogamy. Polyandry individuals also invested more in metabolites associated with increased endurance capacity and efficient energy metabolism and regulation, namely lipids and glycogen. Finally, polyandry males were less desiccation‐ and starvation resistant than monogamy males, suggesting trade‐offs between resistance and sexually selected traits. Our results provide experimental evidence that mating systems can impose selection that influences the evolution of non‐sexual phenotypes such as development, activity, metabolism and nutrient homeostasis.

## Graphical abstract

Sexual selection and sexual conflict are expected to affect all aspects of the phenotype, not only traits that are directly involved in reproduction. Here, we show coordinated evolution of multiple physiological and life history traits in response to long‐term experimental manipulation of the mating system in populations of Drosophila pseudoobscura. Our results provide experimental evidence that mating systems can impose selection that influences the evolution of non‐sexual phenotypes such as development, activity, metabolism, and nutrient homeostasis.

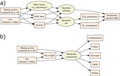

## 1 INTRODUCTION

Natural and sexual selection often act differently in males and females, which have different routes to evolutionary fitness as a result of anisogamy (Darwin, 1871; Kokko & Jennions, 2008). However, independent evolution of the sexes is partly constrained due to their shared genome (Lande, 1980). Recent work has increasingly highlighted that sexual selection affects all aspects of the phenotype, and not only classic sexually selected traits such as male ornaments (reviewed in Cally et al., 2019). This is because sexually selected traits often depend on overall condition (Rowe & Houle, 1996), which in turn depends on underlying aspects of organisms such as their development, physiology, life history and metabolism (Lailvaux & Irschick, 2006; Orr & Garland, 2017).

Sexual selection often favours energetically costly bouts of sustained locomotor activity, for example, during mate searching, courtship or the ‘harassment and resistance’ behaviours that typify interlocus sexual conflict (Debelle et al., 2017; Gyulavári et al., 2014; Hunt et al., 2004; Kotiaho, 2001; Watson et al., 1998). Moreover, postcopulatory sexual selection can select for males that produce metabolically expensive ejaculates (Immonen et al., 2016; Linklater et al., 2007). We therefore expect sexual selection and sexual conflict to favour physiological adaptations that augment the efficiency of metabolism and respiration (Gyulavári et al., 2014; Montooth et al., 2003). Sexual selection is also hypothesized to affect the evolution of development and life history, potentially in a sex‐specific way (Badyaev, 2002; Stångberg et al., 2020). For instance, in populations experiencing heightened sexual selection, male *Drosophila melanogaster* evolved increased activity and courtship frequency and shorter lifespan (Nandy et al., 2013). In a separate experiment, males experiencing heightened sexual selection evolved faster development time, whereas females developed faster under monogamy (Hollis et al., 2017). Likewise, selection for early‐life reproduction in *Acanthoscelides obtectus* beetles favoured higher metabolic rate in males (Arnqvist et al., 2017), and in *Callosobruchus maculatus* beetles, females (but not males) evolved under polygamy had higher mortality and aging rates than females evolved under monogamy (Maklakov et al., 2007). Thus, selection may cause trade‐offs between different aspects of fitness, for instance, favouring a ‘live fast, die young’ strategy in species where competition for mates is intense (Hämäläinen et al., 2018; Hollis et al., 2017; Nandy et al., 2013) or selecting for the reallocation of limiting resources away from growth and somatic maintenance (Berson et al., 2019; Emlen et al., 2012; Hunt et al., 2004; Pitnick et al., 1995). Indeed, the conspicuous fitness trade‐offs associated with sexually selected traits was a key motivation for the theory of sexual selection (Darwin, 1871).

In addition to these links between sexually selected traits and other phenotypes, the picture is further complicated by genetic correlations between the sexes. Male and female traits have a shared genetic basis, such that selection on males results in a (frequently maladaptive) evolutionary response in females, and *vice versa* (e.gArnqvist et al., 2017; Berger et al., 2014; Hämäläinen et al., 2018; Harano et al., 2010; Holman & Jacomb, 2017; Immonen et al., 2018; Jensen et al., 2015; Lewis et al., 2011; Long & Rice, 2007; Videlier et al., 2019). Therefore, sexual selection on males produces an evolutionary response in female, as well as male, life‐history traits and vice versa. For instance, male‐limited selection for short life span in *C*. *maculatus* caused a correlated response in females (Berger et al., 2014), and in *D*. *melanogaster*, male‐limited evolution selected for increased activity in both sexes, to the detriment of female fitness (Long & Rice, 2007). Although these studies have measured the response of individual or several key physiological or life‐history traits to the strength of sexual selection, in this paper, we investigate the effect of variation in the strength of sexual selection on multiple inter‐related life‐history and physiological traits to capture a broader range of consequences of sexual selection.

We use the fruit fly, *Drosophila pseudoobscura*, subjected to either experimentally enforced monogamy (M) that greatly reduces the opportunity for sexual selection and sexual conflict, or to elevated polyandry (E), where 6 males were housed with 1 female to facilitate heightened inter‐ and intra‐ sexual selection. In nature, rates of multiple paternity in *D*. *pseudoobscura* range between 52 and 92% with more than one sire per brood, such that the E treatment greatly increases the opportunity for sexual selection compared to natural populations (Anderson, 1974; Cobbs, 1977; Price et al., 2008). For each sexual selection treatment, there were four replicate populations (hereafter ‘lines’). Previous studies have found divergence between the E and M lines in several traits important during episodes of sexual selection. For example, E males produce more abundant and complex chemical signals (Hunt et al., 2012), perform a faster and more vigorous courtship song (Debelle et al., 2014, 2017; Snook et al., 2005), court and mate more frequently (Crudgington et al., 2010), are more competitive in mating encounters (Debelle et al., 2016) and have larger glands for producing seminal fluid (Crudgington et al., 2009). Additionally, co‐evolutionary patterns have been found, such that E males are more harmful but E females are more resilient to such harm (Crudgington et al., 2005, 2009), and females prefer the courtship song of males from their own treatment (Debelle et al., 2014). Divergence between treatments could be transient. However, previous studies have shown that evolved differences persist and are stable after tens of generations (Debelle et al., 2014, 2017; Snook et al., 2005). Moreover, evolved phenotypic differences are accompanied by divergence in gene expression (Immonen et al., 2014; Veltsos et al., 2017) and concerted genetic differences between treatments that are consistent across replicates (Wiberg et al., 2021). Many genes differentially expressed between treatments are also found within differentiated genomic regions (Wiberg et al., 2021).

Given these evolved differences in traits directly associated with reproduction, we tested whether the mating system similarly caused divergence in life‐history and physiological phenotypes that likely contribute to reproductive success, namely metabolic rate, macrometabolite content, development time and stress resistance. Assuming that these traits contribute to reproductive success, we predict that they may have diverged between the E and M lines, reflecting the different behaviours, life‐history decisions and physiological states that maximize fitness under the different selective regimes. Due to the genetic nonindependence of the sexes, the multiple differences in selection on both sexes in the E and M treatments and the genetic correlations between the variables examined, the direction of evolutionary change is difficult to predict, making our study exploratory in nature. However, we predict higher activity levels and metabolic rates in the E treatment in both sexes, due to the elevated importance of courtship, harassment and resistance behaviours. As such, elevated metabolism might require altered resource allocation, affecting macrometabolite composition, development time and the ability to resist stressors. Shorter development may be favoured by sexual selection (Hollis et al., 2017); however, the effects of sexual selection on macrometabolite profile is unknown *a priori*.

## 2 MATERIALS AND METHODS

### 2.1 Establishment and maintenance of experimental evolution lines

Details of the establishment and maintenance of the experimental evolution lines has been previously described (Crudgington et al., 2005). Briefly, the ancestral population was established from 50 wild‐caught, inseminated female *Drosophila pseudoobscura* from Tucson, Arizona collected in 2001; the descendants of these females were used to establish four replicate populations (termed lines) for each sexual selection treatment. In the enforced monogamy or ‘M’ treatment, the adult population was housed in groups of two (one male, one female); this protocol reduces the opportunity for sexual selection and sexual conflict. In the elevated polyandry or ‘E’ treatment, each group of adults comprised six males and one female. Each M line contained a greater number of groups (*n *= 80) than each E line (*n *= 40), to offset the smaller group size and thereby minimize differences between treatments in the autosomal effective population size (Snook et al., 2009). In each generation, unmated males and females were housed in ‘interaction vials’ (IVs) for 5 days, before being transferred to ‘oviposition vials’ (OVs) for a further 5 days; using both IVs and OVs reduces larval competition and provides more opportunity for episodes of pre‐ and postcopulatory sexual selection. To facilitate selection favouring the most productive groups (and the most competitive males, for the E treatment), offspring from all groups within each line were gathered and mixed in a single container, and a random sample of all the offspring was used to set up the next generation. Flies were kept at 22ºC on a 12:12 light:dark cycle on standard cornmeal–agar–molasses media with added live yeast.

### 2.2 Experimental individuals

Prior to the experiments described below, flies from each line were taken out of selection and placed in a ‘common garden’ for one generation to minimize nongenetic differences between lines. Newly eclosed individuals were collected *en masse* from the OVs within each line. A random sample of these flies were allowed to mate and oviposit for two days. From these eggs, we set up controlled density vials (CDVs) by placing 100 first instar larvae into food vials. Unmated flies eclosing from CDVs were collected and stored in same‐sex food vials until 3–5 days old. Apart from the development time experiment, each measurement in our study was performed on a ‘triad’ of three age‐matched, same sex flies within each line, due to practical constraints resulting from the small size of individual flies when measuring respirometry and metabolite composition. We therefore also measured desiccation and starvation resistance on triads so as to measure traits in a consistent way.

### 2.3 Juvenile development time

We measured juvenile development time at generations 180, 179, 178 and 176 for lines 1–4, respectively. For each replicate of the E and M treatments, we seeded 6 CDVs on three consecutive days (i.e. 600 larvae per replicate population per seeding day = 14,400 larvae). Vials were checked daily for new eclosees, and flies were CO_2_ anaesthetized and killed in ethanol. We continued collecting until no individuals eclosed for two consecutive days. We subsequently counted the number of adult males and females emerging each day from each vial.

We used the length of wing vein four as a proxy for body size (Crudgington et al., 2005) by measuring a random subsample of individuals (*n *= 15 per sex per replicate per seeding day). We removed the left wing from flies preserved in ethanol and mounted wings on a microscope slide in a drop of phosphate‐buffered saline and dried at room temperature overnight. We imaged wings using a Motic camera and Motic Images Plus 2.0 software (Motic Asia, Hong Kong). We measured the length of wing vein four using ImageJ software (Schneider et al., 2012). Image files were anonymized prior to measurement.

### 2.4 Desiccation and starvation resistance

We measured desiccation and starvation resistance at generations 199, 198, 197 and 195 for lines 1–4, respectively. Triads (*n *= 7–10) were housed in 8‐dram plastic vials stoppered with cotton balls and covered with Parafilm^®^. For the desiccation resistance assay, vials contained no food and between the cotton and Parafilm^®^ we placed a packet of silica gel beads. For the starvation resistance assay, vials contained an agar solution that provided moisture but no food. Vials were checked every 2 hours and any deaths were recorded until all flies perished. Flies were scored as dead if they were not able to right themselves or no movement was observed.

### 2.5 Respirometry

We measured metabolic rates at generations 196, 195, 194 and 192 for lines 1–4, respectively. Triads (*n *= 3) were weighed to the nearest 0.1 mg (Sartorius Genius ME 235P‐OCE) before transfer to a respirometry chamber (a glass cylinder; 17mm ×70 mm). Metabolic rate was measured at 19ºC as in Kurbalija Novičić et al. (2015) using a Sable Systems (Las Vegas, NV, USA) respirometry system (Lighton, 2008). This system pumps air at a precise flow rate through a sealed chamber containing the organisms undergoing measurement. Downstream gas analysers measure two response variables: the amount of CO_2_ produced and O_2_ consumed. Briefly, the respirometry system was set up in stop‐flow mode (Lighton, 2008), in which each chamber was sealed for 60 min and then flushed for 2.5 min. Each cycle (through all 24 chambers) lasted for 62.5 minutes, and each triad of flies was recorded over consecutive cycles, giving four readings of CO_2_ and O_2_ flux in each individual chamber. The first recording was discarded as a wash‐out, and the other three were used for analyses. Each respirometry chamber was placed in an activity detector (AD‐2, Sable Systems) connected to a data acquisition interface (Quick‐DAQ, National Instruments, Coleman Technologies, Newton Square, US), which uses reflective infrared light technology to provide a precise and continuous measure of locomotor activity of the subjects; one activity measurement per triad per cycle was recorded. One of the 24 chambers was left empty and used as a baseline to control for any drift of the gas analysers during each session (washed out twice in each cycle). Thus, our measure of metabolic rate is based on the observations of three consecutive readings of the amount of CO_2_ produced and O_2_ consumed during 62.5 minutes by a triad of flies at 19ºC under dark conditions, with a known weight and total amount of activity performed. We also calculated the respiratory quotient (RQ; i.e. VCO_2_ / VO_2_), which describes the metabolic substrate used for respiration, where a value of 0.7 indicates pure fatty acid oxidation, 1.0 indicates pure carbohydrate oxidation and intermediate values indicate mixed substrate or protein‐based oxidation.

### 2.6 Metabolite extractions

We measured metabolite composition at generations 199, 198, 197 and 195 for lines 1–4, respectively. Triads (*n *= 3) were weighed to the nearest 1µg (METTLER TOLEDO^®^ UMX2 ultra‐microbalance) and flash frozen in liquid nitrogen. Triads were then placed in a 0.35‐ml glass vial insert (SUPELCO Analytical^®^) of known weight, dried at 55ºC overnight and reweighed to obtain dry weight. We subsequently processed each sample to acquire measurements of lipids, soluble carbohydrates, soluble protein, glycogen and chitin (see Supporting information).

### 2.7 Statistical analysis

All statistical analyses were performed in R version 4.0.3 (R Core Team, 2020). We fit models using Bayesian approaches in *brms* (Bürkner, 2017), and specified conservative, regularizing priors on the fixed and random effects. Complete code and description of models are provided in the code repository (https://lukeholman.github.io/exp_evol_respiration/). In brief, we analysed development time and survival using survival analysis. For the respirometry and metabolite data, we used structural equation models (SEM), motivated by the causal relationships that we hypothesize to exist between the variables, given the experimental design and our biological understanding of the system (Figure S1). For the metabolite data, we began by expressing the abundance of each metabolite as a proportion of dry weight and scaled each response variable to have mean zero and unit standard deviation. For the SEMs, after fitting the model, we calculated the treatment effect size (Cohen's *d*) for each response variable, both with and without the moderators (dry weight and/or activity). We also calculated the difference in treatment effect size between the sexes, to test for a treatment × sex interaction. All models included replicate line as a random intercept to reflect the experimental design of the selection experiment (i.e. where *n *= 8). We also performed analyses with selection treatment fitted as a random slope to allow lines to vary in their response to selection (Arnqvist, 2020; Schielzeth & Forstmeier, 2009); however, results were qualitatively identical, and thus we present the results of models with random intercepts only in the Results (see Supporting information for results including random slopes). All models also included vial identity as a random intercept, except for wing vein length where we measured a random sample of individuals across multiple vials. To provide a quantity somewhat analogous to a frequentist p‐value, we calculated the posterior probability that the true effect size is zero or of the opposite sign to that reported. The posterior probability (PP) tends to zero as we become increasingly certain of the direction of the effect.

## 3 RESULTS

### 3.1 Juvenile development time

**FIGURE 1:**
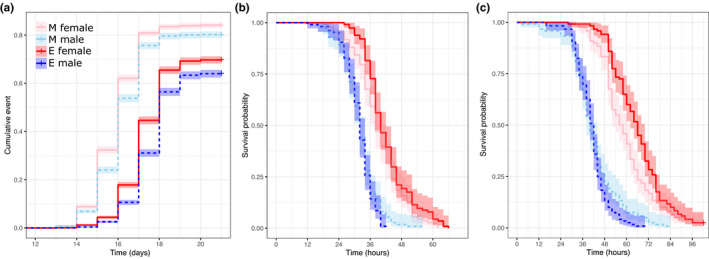
Kaplan–Meier plots for (a) juvenile development time, (b) desiccation resistance and (c) starvation resistance. ‘+’ indicates right censored individuals. Monogamy (M) = pink and light blue; elevated polyandry (E) = red and dark blue; males = dashed lines; females = solid lines. Shaded areas show confidence intervals

Juvenile development time differed significantly between the E and M treatments (Cox proportional hazards model; PP = 0.008) and between the sexes (PP < 0.001). E treatment flies had a reduced hazard (Hazard ratio = 0.44; 95% confidence intervals [CI] = 0.26–0.81), that is, development took longer in the E treatment in both sexes. Males took longer to eclose than females (Hazard ratio = 0.84; 95% CI = 0.80–0.88) (Figure 1a), as usual in this species. There was a significant treatment × sex interaction (PP = 0.007), such that the sex difference in development time was greater in the E treatment, and the treatment affected males more strongly than females.

As expected, females were larger than males (mean wing vein four length (µm); M females: 2324 ± 4.80, *n *= 152; E females: 2335 ± 5.76, *n *= 118; M males: 2099 ± 4.93, *n *= 154; E males: 2114 ± 5.96, *n *= 127). We found no statistically significant effect of sexual selection treatment (PP = 0.312) or the treatment × sex interaction (PP = 0.382).

### 3.2 Desiccation and starvation resistance

We found a significant treatment × sex interaction for both desiccation and starvation resistance (desiccation: PP = 0.024; starvation: PP = 0.018). E males survived 0.91 (95% CI = 0.83–0.99) and 0.88 (95% CI = 0.80–0.97) times as long as M males under desiccation and starvation resistance, respectively (Figure 1b,c). However, the treatment effect was opposite in females: E females survived 1.06 (95% CI = 0.87–1.29) and 1.09 (95% CI = 0.88–1.35) times longer than M females under desiccation and starvation resistance, respectively. In short, M males survived longer than E males, whereas E females survived longer than M females.

### 3.3 Respirometry

**FIGURE 2:**
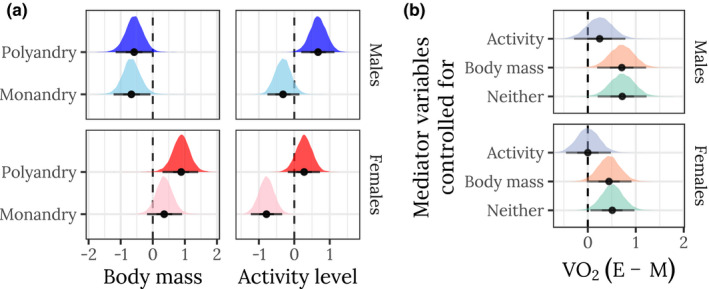
Effects of sex and selection treatment on metabolic rates and mediator variables. a) Posterior estimates of the means for mediator variables (body mass and activity). Note that females are larger than males, and that E females are somewhat larger than M females. b) Posterior estimates of effect sizes of selection treatment (E minus M) on metabolic rate (measured as volume of O2 consumed) controlling for activity, body mass or neither mediator variable. The x‐axes show the posterior estimate of standardized effect size (Cohen's d), that is, a value of 1 would mean that the E treatment has a mean that is larger by 1 standard deviation. The horizontal bars show the 66% and 95% quantiles and the median of the posterior distribution. All response variables have been mean‐centred and divided by the overall standard deviation (such that the dashed line at zero marks the mean across sexes, treatments and cycles). For brevity, only the first cycle is shown for activity and metabolic rate; see Supporting information for all cycles

Beginning with the mediator variables, females were heavier than males (PP < 0.001; Table S1), but there was no significant effect of sexual selection treatment (PP = 0.075) or the treatment × sex interaction (PP = 0.105; Figure 2a; mean dry weight per triad (mg): M females: 0.40 ± 0.02; E females: 0.47 ± 0.04; M males: 0.29 ± 0.02; E males: 0.29 ± 0.02; *n *= 12 each). There was a significant effect of treatment on activity level (PP < 0.001), with E flies more active than M flies (Figure 2a). Activity level also showed a sex × cycle interaction (PP < 0.026), indicating that activity levels declined over cycles in males but not females.

There was a strong correlation between activity level and O_2_ consumption (PP < 0.001; Figure S2), and O_2_ consumption declined across cycles (PP < 0.001). Body weight did not correlate with O_2_ consumption (PP = 0.119; Figure S2). Calculating the posterior estimate of the difference in treatment means for each sex and cycle revealed that O_2_ consumption was higher in the E treatment (indicated by positive effect sizes in Figure 2b), especially in males. This effect was considerably attenuated when we statistically adjusted for differences in activity level between treatments and sexes, indicating that the evolved difference in activity between the E and M treatments was largely responsible for the treatment effect on O_2_ consumption. Controlling for the evolved differences in body mass did not change the effect size, illustrating that the (modest) changes in body mass between the E and M treatments did not explain the evolved difference in O_2_ consumption.

The respiratory quotient (RQ) did not differ detectably between the E and M treatments, whether or not one controlled for the mediator variables (Figure S3). The grand mean RQ = 0.90 (± 0.02) indicated flies used a mixture of carbohydrates, protein and lipids as metabolic substrate.

### 3.4 Metabolite composition

Females were heavier than males (PP < 0.001) and E flies were heavier than M flies (PP = 0.002; dry weight (mg): M females: 0.56 ± 0.02; E females: 0.64 ± 0.17; M males: 0.33 ± 0.01; E males: 0.35 ± 0.01; *n *= 12 each; Table S2). There was a significant treatment × sex interaction (PP = 0.038), such that the E treatment positively affected body mass more strongly in females. Furthermore, dry weight was significantly correlated with lipid and chitin content (Figure S4; Table S2) and, therefore, could act as a mediator for some of the effect of sex and treatment on these metabolites.

**FIGURE 3:**
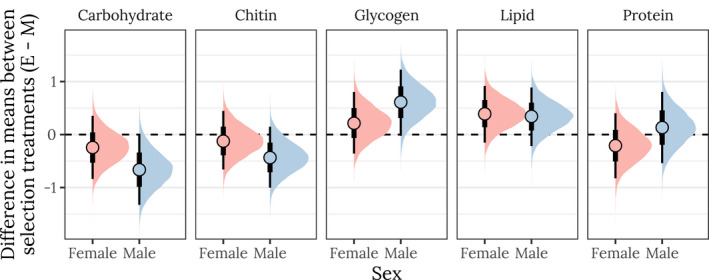
Posterior estimates of the treatment effect size for both sexes, for each of the five metabolites. A positive value indicates that the mean metabolite content is higher in the E treatment than the M treatment, whereas a negative value denotes M > E. A strongly supported treatment effect is implied by most of the posterior lying to one side of zero. The vertical bars show the 66% and 95% quantiles and the median of the posterior distribution. This plot was created using posterior predictions of the means that were adjusted for differences in dry weight between treatments

We also found differences in metabolite composition between the E and M treatments and between sexes. M treatment males had more carbohydrates and chitin, whereas E males had more glycogen. E treatment females had more lipids than M females (Figure S5). When we controlled for the evolved differences in dry weight between the E and M treatments, only the carbohydrate and glycogen differences between E and M males remained statistically significant (Figure 3); however, the change in effect size when controlling for dry weight was very small, indicating that dry weight was not an especially important mediator of the evolved changes in metabolite composition (compare Figure 3 and Figure S5). Finally, we investigated the treatment × sex interaction term by calculating the posterior difference in the treatment effect size between sexes but found no significant differences in effect sizes between the sexes (Figure S6).

## 4 DISCUSSION

We found that, in addition to reproductive traits, experimental manipulation of sexual selection and sexual conflict caused several physiological and life‐history traits to evolve. First, flies from the elevated polyandry (E) treatment took longer to develop into adults than their monogamous (M) counterparts and the difference in development time between the sexes was greater in the E treatment. Second, both E males and females had faster metabolic rates, largely due to increased activity compared to M males and females. Third, macrometabolite composition has diverged between sexual selection treatments, such that E females had a greater lipid content than M females, E males greater glycogen content than M males and E males less chitin and sugar content than M males. Finally, E males were less resistant to desiccation and starvation than M males, whereas E females were more resistant than M females.

E males produce a faster and more vigorous courtship song (Debelle et al., 2014, 2017), more cuticular hydrocarbons (Hunt et al., 2012) and larger accessory glands (Crudgington et al., 2009) than M males. These sexually selected traits may be energetically costly to produce and maintain (Debelle et al., 2017), demanding greater metabolic activity (Berson et al., 2019; Immonen et al., 2016; Montooth et al., 2003). Although selection for early‐life reproduction resulted in a sex‐specific response of metabolic rate in *A*. *obtectus* beetles (Arnqvist et al., 2017), in *D*. *melanogaster*, both locomotor activity and metabolic rate have a significant intersexual genetic correlation (Long & Rice, 2007; Nandy et al., 2013; Videlier et al., 2021). Therefore, sexual selection favouring increased activity and metabolic rate in males may generate sexual conflict by increasing activity and metabolic rate above optimal levels for females (Hämäläinen et al., 2018). In *D*. *pseudoobscura*, we found E flies had higher metabolic rates in both sexes, which was largely explained by evolved differences in activity level, suggesting the potential for sexual conflict over metabolic rate. However, we found no difference between treatments or sexes in the respiratory quotient (RQ), which describes metabolic substrate use, despite evolved differences in activity and metabolic rate. Increased energy expenditure may require a shift toward more energy‐dense fuel for respiration and a resultant shift in RQ. The lack of divergence of RQ between treatments suggests that E flies did not evolve to use different ratios of macronutrients for respiration alongside their elevated activity levels.

We found that E males had higher glycogen content than M males. Carbohydrates are the main fuel used during intense aerobic activities, such as flight (Wigglesworth, 1949) and courtship (Bertram et al., 2011), and glycogen provides the main source of trehalose marshalled during intense activity (Becker et al., 1996). Sexual selection favouring endurance capacity may also increase lipid respiration and fat storage (Gyulavári et al., 2014). However, despite male courtship song being an endurance contest (Debelle et al., 2017), in males we found no difference between treatments in lipid content. Limited time and resources may demand strategic resource allocation decisions. We did find higher lipid content in E females, possibly reflecting selection for endurance capacity, as E females are courted more frequently (Crudgington et al., 2005; Debelle et al., 2014). None of the metabolites showed a significant treatment × sex interaction, indicating the response to sexual selection treatment did not differ between the sexes, potentially highlighting a strong intersexual genetic correlation constraining divergence of physiological traits (Videlier et al., 2021; Wittman et al., 2021). Our results suggest that heightened sexual selection and sexual conflict favours storage and regulation of metabolites to meet increased metabolic demands (Crudgington et al., 2009; Debelle et al., 2014, 2017; Gyulavári et al., 2014; Montooth et al., 2003), whereas relaxed sexual selection may instead enable investment in other components of fitness. Studies manipulating diet (e.g. nutritional geometry framework) will help inform our understanding of potential trade‐offs and allocation decisions affecting different life‐history traits under sexual selection (Gray et al., 2018; Morimoto & Wigby, 2016).

E males were less tolerant of desiccation and starvation than M males despite their greater glycogen content, which buffers against these stresses (Marron et al., 2003). Previous work artificially selecting on desiccation tolerance in *D*. *melanogaster* found selected lines had lower metabolic rates (Hoffmann & Parsons, 1989); our experiment imposed different selective pressures but found the same negative correlation between metabolic rate and stress resistance. The E lines have greater cuticular hydrocarbon (CH) content but there is no difference in the abundance of long‐chain CHs (which offer greater desiccation resistance) between treatments (Hunt et al., 2012). That E males are less stress resistant despite having more macromolecules which buffer against such stressors suggests a trade‐off, where heightened sexual selection favours investment in current reproduction at a cost to later life survival, whereas the relaxation of sexual selection favours increased investment in longevity (Hunt et al., 2004; Kotiaho, 2001; Nandy et al., 2013). In contrast, E females showed no such trade‐off, as E females were as stress resistant as M females, despite investing more in fecundity (Crudgington et al., 2005; Immonen et al., 2014). The greater lipid content we found in E females could mitigate such a trade‐off, because lipids provide protection against starvation alongside being a major component of eggs (Chippindale et al., 1996). Thus, the sexes have altered investment in macrometabolite composition under sexual selection, which may allow differential investment in sexually selected traits and sexual conflict persistence and resistance traits. The subsequent fitness consequences during stress have different outcomes on the sexes. This suggests sex‐specific selection in the adult stage which could further generate sexual conflict over shared traits, particularly during the juvenile stage (Badyaev, 2002).

Related to the juvenile stage, in both sexes, E flies took significantly longer to reach adulthood than M flies. The shared response is likely due to development time probably being genetically correlated across sexes (Berger et al., 2014; Lewis et al., 2011). However, whether longer development time benefits both sexes or is under sexual conflict is unclear. For example, E males may require longer juvenile development to acquire more resources used for investment in adult sexual traits (e.g. increased activity to pursue females for mating; Crudgington et al., 2009) and E females may similarly benefit in that additional resources acquired could be allocated towards reproduction or resistance to male harm (Crudgington et al., 2005, 2010; Immonen et al., 2014). Alternatively, male development time may be extended under strong sexual selection and sexual conflict to allocate resources to, for example, sex‐specific tissues such as accessory glands whose contents have profound consequences on female reproductive behaviour (Sirot et al., 2015) with pleiotropic effects that may be negative on female development time. In the stalk‐eyed fly, *Cyrtodiopsis dalmanni*, larger accessory glands increase male reproductive success via increased mating frequency but increased size delays posteclosion reproductive maturity (Baker et al., 2003). We have previously demonstrated that E males have larger accessory glands with greater mating capacity and that this increased capacity generates sexual conflict (Crudgington et al., 2009).

Our study used only virgin flies. However, mating causes significant changes in postmating physiology (Videlier et al., 2019) and lifespan (Chapman et al., 1995), and changes in gene expression after mating differ between the E and M treatments (Veltsos et al., 2017). Future studies should investigate how mating alters physiological responses under differing sexual selection regimes. Furthermore, more research is needed to investigate the connection between sexually selected traits and the whole organismal physiological and life‐history traits that support their expression. For example, experiments tracking individual development, physiology, reproductive success and survivorship under different sexual selection treatments will provide a more integrated understanding of the effects of sexual selection on life histories, from divergence in gene regulation to phenotypic responses. How these differences translate to the evolution of whole organism performance in nature are also needed (Noble et al., 2021).

To conclude, we have shown that experimental manipulation of sexual selection and sexual conflict caused the evolution of several traits that are not directly involved in sexual interactions. Differences in activity levels and metabolic rate coincided with differential investment in macrometabolites, reflecting the energetic demands associated with the elevation or removal of sexual selection. Males evolving under elevated polyandry suffered reduced survival under desiccation and starvation stress, potentially trading off current versus future reproduction. However, the same was not true of females, perhaps due to selection favouring endurance capacity in females to offset the costs of frequent male courtship. The physiological and life‐history traits we measured likely have a shared genetic basis, and thus may be subject to intralocus sexual conflict. The magnitude of any sexual dimorphism did not differ between treatments for the physiological traits we measured (macrometabolites and metabolic rate), whereas life‐history traits (development time and stress resistance) were more dimorphic in populations evolving under elevated polyandry, suggesting tighter intersexual genetic correlations over physiological traits. Overall, our findings highlight that sexual selection results in coordinated evolution of fundamental physiological and nonreproductive life‐history traits, implicating sexual selection as an important factor in the evolution of life‐history strategies.

## CONFLICT OF INTERESTS

The authors have no conflict of interest to declare.

## AUTHOR CONTRIBUTIONS

RRS designed the experiments. ALB, MDG, ZKN and RRS collected the data. MDG and LH performed statistical analyses. MDG, LH and RRS wrote the manuscript. All authors agreed to the final version of the manuscript.

### PEER REVIEW

The peer review history for this article is available at https://publons.com/publon/10.1111/jeb.14003.

### OPEN RESEARCH BADGES

This article has been awarded Open Materials, Open Data, Badges. All materials and data are publicly accessible via the Open Science Framework at https://doi.org/10.5061/dryad.9cnp5hqhk and https://lukeholman.github.io/exp_evol_respiration/.

## Supporting information

Supplementary Material

## Data Availability

Data and code are available from the Dryad digital repository: https://doi.org/10.5061/dryad.9cnp5hqhk, and GitHub: https://lukeholman.github.io/exp_evol_respiration/
